# Meningioma MRI radiomics and machine learning: systematic review, quality score assessment, and meta-analysis

**DOI:** 10.1007/s00234-021-02668-0

**Published:** 2021-03-02

**Authors:** Lorenzo Ugga, Teresa Perillo, Renato Cuocolo, Arnaldo Stanzione, Valeria Romeo, Roberta Green, Valeria Cantoni, Arturo Brunetti

**Affiliations:** 1 Department of Advanced Biomedical Sciences, University of Naples “Federico II”, Via Pansini 5, 80131 Naples, Italy; 2 Department of Clinical Medicine and Surgery, University of Naples “Federico II”, Via Pansini 5, 80131 Naples, Italy

**Keywords:** Systematic review, Meta-analysis, Machine learning, Meningioma, Magnetic resonance imaging

## Abstract

**Purpose:**

To systematically review and evaluate the methodological quality of studies using radiomics for diagnostic and predictive purposes in patients with intracranial meningioma. To perform a meta-analysis of machine learning studies for the prediction of intracranial meningioma grading from pre-operative brain MRI.

**Methods:**

Articles published from the year 2000 on radiomics and machine learning applications in brain imaging of meningioma patients were included. Their methodological quality was assessed by three readers with the radiomics quality score, using the intra-class correlation coefficient (ICC) to evaluate inter-reader reproducibility. A meta-analysis of machine learning studies for the preoperative evaluation of meningioma grading was performed and their risk of bias was assessed with the Quality Assessment of Diagnostic Accuracy Studies tool.

**Results:**

In all, 23 studies were included in the systematic review, 8 of which were suitable for the meta-analysis. Total (possible range, −8 to 36) and percentage radiomics quality scores were respectively 6.96 ± 4.86 and 19 ± 13% with a moderate to good inter-reader reproducibility (ICC = 0.75, 95% confidence intervals, 95%CI = 0.54–0.88). The meta-analysis showed an overall AUC of 0.88 (95%CI = 0.84–0.93) with a standard error of 0.02.

**Conclusions:**

Machine learning and radiomics have been proposed for multiple applications in the imaging of meningiomas, with promising results for preoperative lesion grading. However, future studies with adequate standardization and higher methodological quality are required prior to their introduction in clinical practice.

**Supplementary Information:**

The online version contains supplementary material available at 10.1007/s00234-021-02668-0.

## Introduction

Meningiomas are the most common primary intracranial tumor in adults, being more frequent in middle-aged women [1]. The average age-adjusted yearly incidence rate is 7.86 cases per 100.000 individuals, which has increased during the past 30 years due to the improvement of diagnostic imaging [2]. Magnetic resonance imaging (MRI) is the modality of choice for their radiological diagnosis and follow-up, whereas computed tomography (CT) is used when patients cannot undergo MRI. The World Health Organization (WHO) classification of central nervous system tumors of 2016 grades meningiomas into three groups: grade I (slowly growing tumors), grade II (atypical meningioma), and grade III (anaplastic or malignant meningioma) [3]. Among these, grade II and III meningiomas are associated with high rates of recurrence and premature mortality [4]. Although conventional imaging is usually reliable for meningioma evaluation, it still presents some limitations, in particular in determining pathological grading from preoperative scans [5].

The term radiomics includes different quantitative radiological image analysis techniques, ranging from first order statistics to texture analysis [6]. These produce large amounts of data that can be challenging to process with classical statistical methods but may contribute novel imaging biomarkers. Machine learning (ML), a subfield of artificial intelligence, has seen growing interest in medicine and especially in radiology for numerous applications [7–10]. In particular, supervised learning, based on labeling of data by an expert, is mainly employed for classification and regression tasks. Among the promises of ML for clinical practice, there are automatic detection and characterization of lesions and the possibility to predict response to therapy and risk of recurrence [11–13]. Regarding neuroradiology, it has shown good results in different applications, especially in the field of neuro-oncology [14–16]. In recent years, the number of investigations based on these techniques published allows for data pooling potentially achieving higher levels of evidence through systematic reviews and/or meta-analyses.

Aim of this systematic review is to analyze the methodological quality of prospective and retrospective studies published on radiomics analyses of intracranial meningiomas. Furthermore, a meta-analysis of those employing ML algorithms for the MRI preoperative assessment of meningioma grading has been performed.

## Materials and methods

### Literature search

The PRISMA-DTA (Preferred Reporting Items for Systematic Reviews and Meta-analysis for Diagnostic Test Accuracy) statement was used for this systematic review [17]. Primary publications in English using radiomics and/or ML in MRI exams of meningioma patients, published between 01/01/2000 and 30/06/2020, were searched for on multiple electronic databases (PubMed, Scopus, and Web of Science). The search terms consisted of machine learning OR artificial intelligence OR radiomics OR texture AND meningioma; the detailed search string is presented in the supplementary materials.

Two researchers determined the eligibility of the articles though title and abstract evaluation. Case reports, non-original investigations (e.g., editorials, letters, reviews), and studies not focused on the topic of interest were excluded. The full text of articles in which radiomics was employed on CT or MRI images of intracranial meningiomas were obtained for further evaluation. The reference lists of included studies were also screened for potentially eligible articles and those evaluating the grading of meningioma through ML were selected to perform a meta-analysis.

### Data collection and study evaluation

The radiomics quality score (RQS) was used to evaluate the methodological quality of the studies included in the systematic review whereas the Quality Assessment of Diagnostic Accuracy Studies (QUADAS-2) was used to assess the risk of bias of the studies included in the meta-analysis [18, 19]. For studies included in the meta-analysis, the predictive accuracy was quantified using the AUC for the receiver operator characteristic (ROC) analysis [20]. The number of low (grade I) and high (grade II–III) lesions used to test the model, the source of the dataset, MRI sequences employed to extract the features, ML algorithm, and type of validation were also recorded.

The RQS is a tool developed to assess the methodological quality of studies using radiomics. It evaluates image acquisition, radiomics features extraction, data modeling, model validation, and data sharing. Each of the 16 items it comprises is rated, and the summed total score ranges from −8 to 36, converted to a percentage score where −8 to 0 is defined as 0% and 36 as 100% [18] (Table 1). Three readers with previous experience in radiomics independently assigned an RQS score to each article included in the systematic review.

**Table 1:** Overview of radiomics quality score items and mode of the respective scores in the reviewed studies

RQS checkpoint	RQS item number and name	Description and (points)	Mode
First	Item 1: image protocol quality	Well-documented protocol (+1) AND/OR publicly available protocol (+1)	1
Second	Item 2: multiple segmentation	Testing feature robustness to segmentation variability, e.g., different physicians/algorithms/software (+1)	0
	Item 3: phantom study	Testing feature robustness to scanner variability, e.g., different vendors/scanners (+1)	0
	Item 4: multiple time points	Testing feature robustness to temporal variability, e.g., organ movement/expansion/shrinkage (+1)	0
Third	Item 5: feature reduction	Either feature reduction OR adjustment for multiple testing is implemented (+ 3); otherwise, (−3)	3
	Item 6: multivariable analysis	Non-radiomic feature are included in/considered for model building (+1)	0
	Item 7: Biological correlates	Detecting and discussing correlation of biology and radiomic features (+1)	0
	Item 8: cut-off analysis	Determining risk groups by either median, pre-defined cut-off, or continuous risk variable (+1)	0
	Item 9: discrimination statistics	Discrimination statistic and its statistical significance are reported (+ 1); a resampling technique is also applied (+1)	2
	Item 10: calibration statistics	Calibration statistic and its statistical significance are reported (+ 1); a resampling technique is also applied (+1)	0
	Item 11: prospective design	Prospective validation of a radiomics signature in an appropriate trial (+7)	0
	Item 12: validation	Validation is missing (−5) OR internal validation (+2) OR external validation on single dataset from one institute (+3) OR external validation on two datasets from two distinct institutes (+4) OR validation of a previously published signature (+4) validation is based on three or more datasets from distinct institutes (+5)	2
	Item 13: comparison to “gold standard”	Evaluating model’s agreement with/superiority to the current “gold standard” (+2)	0
	Item 14: potential clinical application	Discussing model applicability in a clinical setting (+2).	2
	Item 15: cost-effectiveness analysis	Performing a cost-effectiveness of the clinical application (+1)	0
	Item 16: open science and data	Open source scans (+1) AND/OR open source segmentations (+1) AND/OR open source code (+1) AND/OR open source representative features and segmentations (+1)	0

*RQS* radiomics quality score

The QUADAS-2 evaluates the risk of bias in different domains (“patient selection,” “index test,” “reference standard,” and “flow and timing”) and can be personalized according to the specific research question [21]. It was assessed in consensus by two readers for each of the studies selected for the meta-analysis.

### Statistical analysis

Continuous variables are presented as mean ± standard deviation. Following previous experiences both with RQS and other scoring systems [22, 23], inter-reader reproducibility was evaluated by calculating the intraclass correlation coefficient (ICC) for the total RQS score obtained by each study. In accordance with recent guidelines, a two-way, random-effects, single-rater, absolute agreement ICC model was used [24]. For the remaining descriptive statistics, RQS score assigned by the most expert reader is reported.

Regarding the meta-analysis, the AUC standard error was calculated from the total number of positive and negative meningiomas patients. The *I*^2^ value was used to assess statistical heterogeneity, providing an estimate of the percentage of variability among included studies. *I*^2^ values of 0–25%, 25–50%, 50–75%, and >75% represent very low, low, medium, and high heterogeneity, respectively. The *I*^2^ statistic describes the percentage of variation across studies that is due to heterogeneity rather than chance [25]. *I*^2^ was calculated as follows: *I*^2^ = 100% × (Q − df)/Q. The weight of each study was calculated with the inverse variance method [26]. The results from all included studies were pooled, and an overall estimate of effect size was evaluated using a random effect model. This approach helped in reducing heterogeneity among studies. Publication bias was examined using the effective sample size funnel plot described by Egger et al. [27]. Two-sided *p* values ≤ 0.05 were considered statistically significant.

The described statistical analyses were performed using R (v3.6.2, “irr” and “auctestr” packages) and MedCalc Statistical Software (version 16.4.3, Ostend, Belgium; https://www.medcalc.org) [28].

## Results

### Literature search

**Fig. 1:**
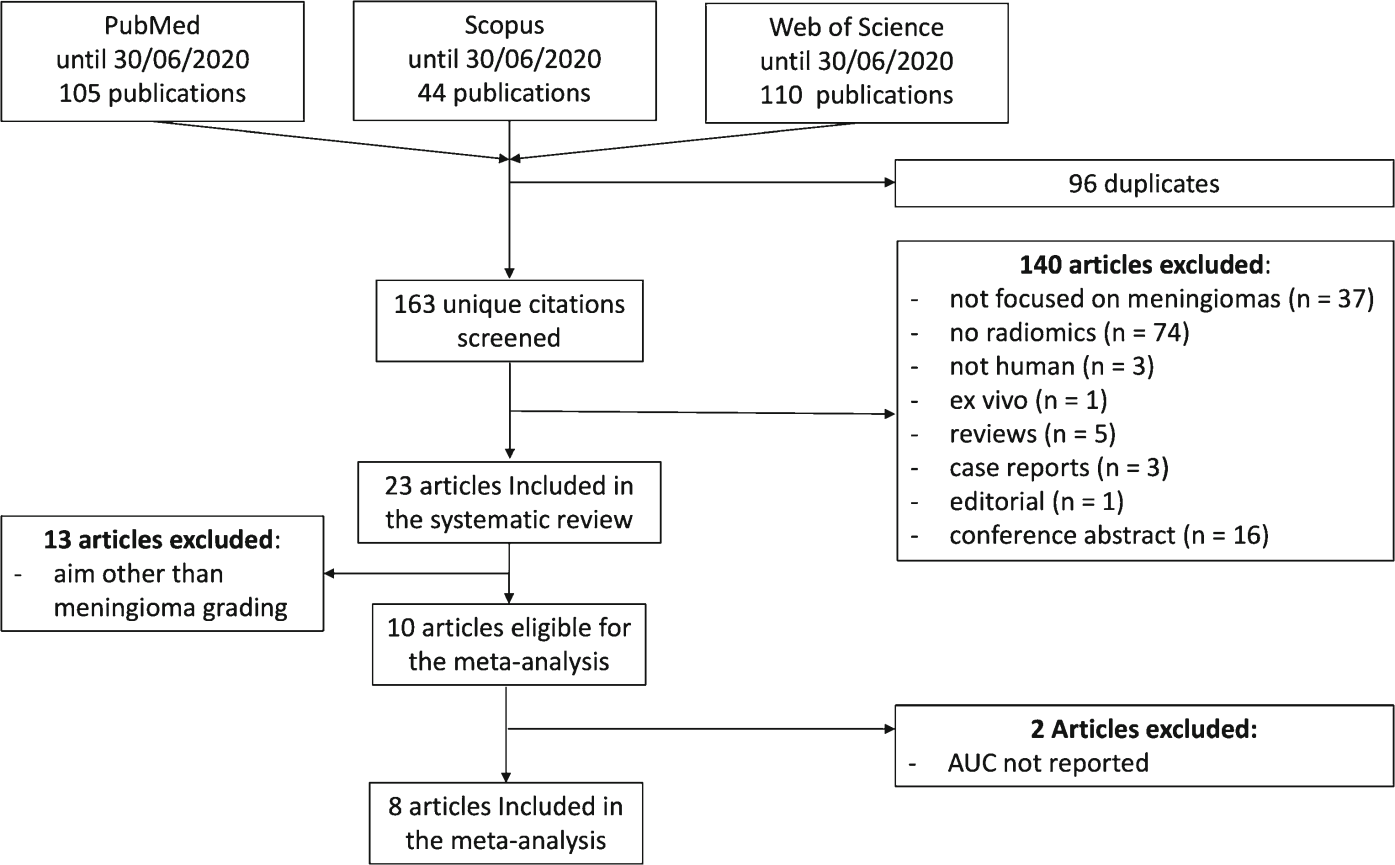
Study selection process flowchart

In total, 256 articles were obtained from the initial search, of which 96 were duplicates. Of the remaining 163, 140 were rejected based on the selection criteria. Finally, 23 articles were included in the systematic review, 8 of which were eligible for the meta-analysis. The described flowchart is represented in Fig. 1, whereas Table 2 contains details on study aim, ML method, and performance.

**Table 2:** Overview of study aim, ML method, and performance for the included studies

Authors	Study aim	ML methodology	Performance
AlKubeyyer et al. 2020 [29]	Development of a computer-aided detection of the meningioma tumor firmness	• Support vector machine • k-nearest neighbor	• F-score=0.95 • Balanced accuracy= 0.87 • AUC=0.87
Arokia Jesu Prabhu et al. 2018 [30]	Automatic classification of parasagittal meningioma	Support vector machine	Accuracy= 0.92
Chen et al. 2019 [31]	Automatic classification of meningiomas	• Linear discriminant analysis • Support vector machine	Accuracy=0.76
Chu et al. 2020 [11]	Prediction of meningiomas grade	Logistic regression	• Accuracy= 0.95 (training group) and 0.93 (test group) • Sensitivity= 0.94 training group) and 0.92 (test group)
Florez et al. 2018 [32]	Differentiation of vasogenic from tumor cell infiltration edema for radiotherapy	Linear regression	AUC>0.71
Hamerla et al. 2019 [33]	Differentiation of low grade from high grade meningioma	• Random forest • Extreme gradient boosting • Support vector machine • Multilayer perceptron	AUC= 0.97 (Extreme gradient boosting)
Kanazawa et al. 2018 [34]	Distinction of solitary fibrous tumor/hemangiopericytoma from angiomatous meningioma	Texture analysis	• Positive predictive value=0.63 • Specificity=0.63
Ke et al. 2019 [35]	Differentiation between benign and non-benign meningiomas	• Support vector machine	• AUC= 0.91 • Accuracy= 0.89 • Sensibility=0.93 • Specificity=0.87
Laukamp et al. 2018 [36]	Automatic detection and segmentation of meningioma	Deep learning	• Detection accuracy=0.98 • Mean Dice coefficient for total tumor volume =0.81 ± 0.10
Laukamp et al. 2019 [37]	Prediction of meningioma grade	Multivariate logistic regression model	AUC=0.91
Li et al. 2019 [38]	Automatic differentiation of malignant hemangiopericytoma from angiomatous meningioma	Texture analysis	AUC=0.90
Lu et al. 2018 [39]	Prediction of meningioma grade using ADC maps	• Classic decision tree • Conditional inference • Decision forest	Accuracy= 0.62
Morin et al. 2019 [40]	Prediction of meningioma grade, local failure and overall survival	Random forest	• Grade= Accuracy 0.65; AUC 0.71 • Local Failure= Accuracy 0.61, AUC=0.68 • Overall Survival= accuracy 0.67, AUC= 0.75
Niu et al. 2019 [41]	Differentiation of meningioma subtypes	Fisher discriminant analysis	Accuracy= 0.99-0.1
Park et al. 2018 [42]	Prediction of grade and histological subtype	• Support vector machine • Random forest	AUC= 0.86
Speckter et al. 2018 [13]	Prediction of response after radiosurgery	Texture analysis	Correlation coefficient=−0.64
Tian et al. 2020 [43]	Contrastive analysis between craniopharyngioma and meningioma	Binary logistic regression	AUC>0.70
Wei et al. 2020 [44]	Differentiation of hemangiopericytoma from meningioma	Logistic regression model	AUC= 0.92–0.99
Yan et al. 2017 [45]	Prediction of meningioma grade	• Logistic regression • Naïve Bayes • Support vector machine	• AUC= 0.73–0.88 • Sensitivity= 0.48–0.91 • Specificity= 0.70–0.96
Zhang et al. 2019 [12]	Prediction of recurrence in skull base meningiomas	Random forest	Accuracy= 0.90
Zhang et al. 2020 [46]	Discrimination of lesions located in the anterior skull base	• Linear discriminant analysis • Support vector machine • Random forest • Adaboos • K-nearest neighbor • GaussianNB • Logistic regression • gradient • boosting decision tree • Decision tree	AUC>0.80
Zhu et al. 2019 [47]	Automatic prediction of meningioma grade	Convolutional neural network	AUC= 0.83
Zhu et al. 2019 [48]	Automatic prediction of meningioma grade	Deep learning	• AUC= 0.81 • Sensitivity= 0.8 • Specificity=0.9

*AUC* area under the receiver operating characteristic curve

### Study evaluation

**Fig. 2:**
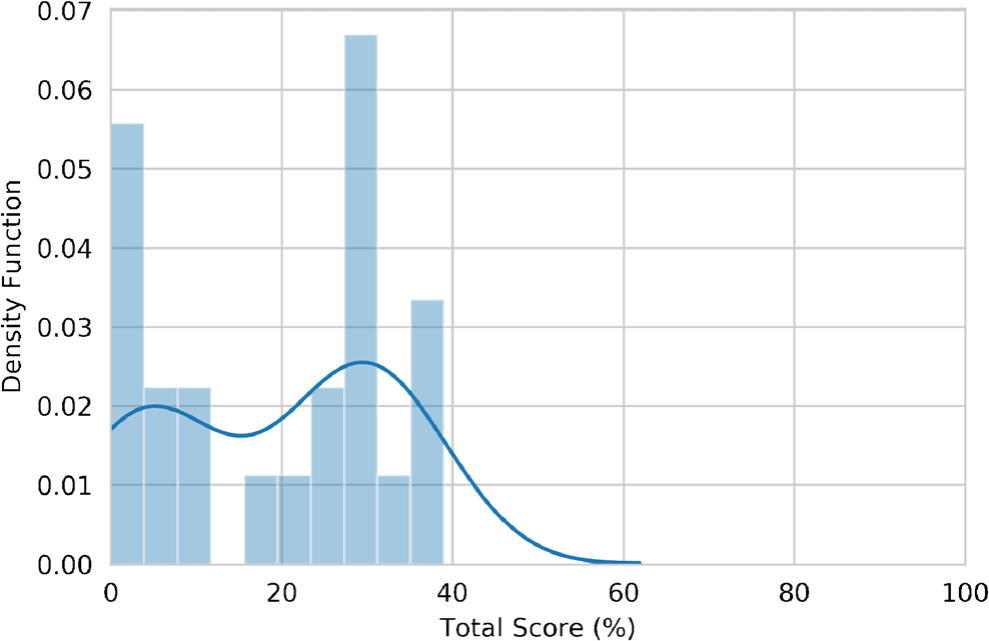
Histogram (bars, bin number = 10) and kernel density estimation (line) plot of RQS percentage score distribution

**Fig. 3:**
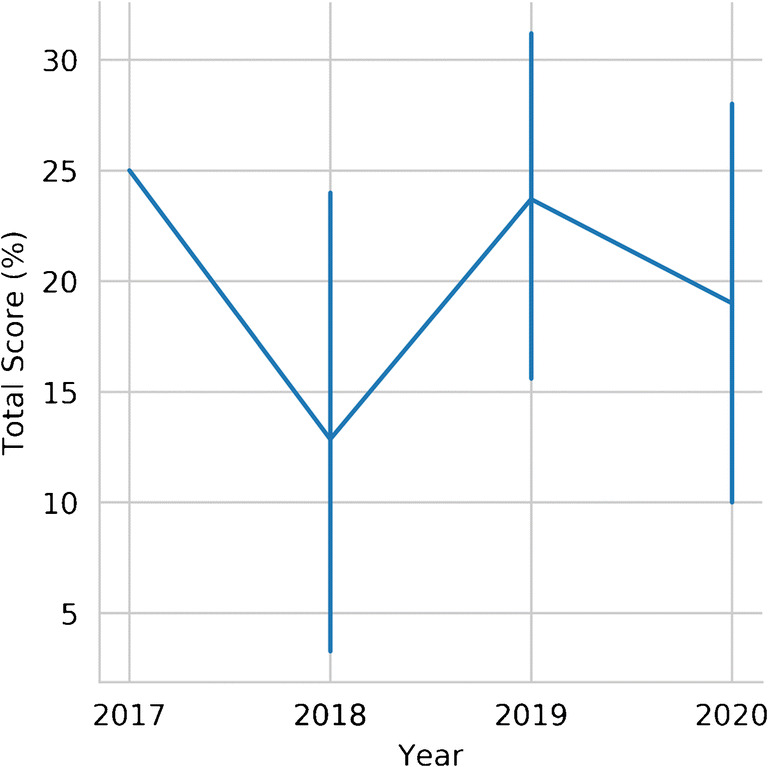
RQS percentage score line plot in relation to publication year. Bars represent 95% confidence intervals, calculated with bootstrapping (1000 iterations)

The RQS total and percentage scores were respectively 6.96 ± 4.86 and 19 ± 13% (Figs. 2, 3). A detailed report of the RQS item score by the most expert reader is shown in Table 3. Inter-reader reproducibility resulted moderate to good, with an ICC = 0.75 (95% confidence intervals, 95% CI = 0.54–0.88). RQS scores assigned by the other readers are presented in the supplementary materials.

**Table 3:** Radiomics quality scores for all included articles

First author	Year	Item 1	Item 2	Item 3	Item 4	Item 5	Item 6	Item 7	Item 8	Item 9	Item 10	Item 11	Item 12	Item 13	Item 14	Item 15	Item 16	RQS (total)	RQS (%)
Alkubeyyer	2020	0	0	0	0	−3	0	0	0	2	0	0	2	0	2	0	0	3	8
Arokia Jesu Prabhu	2018	0	0	0	0	−3	0	0	0	0	0	0	2	0	0	0	0	0	0
Chen	2019	1	0	0	0	3	0	1	0	1	0	0	2	0	2	0	0	10	28
Chu	2020	1	0	0	0	3	0	1	0	1	0	0	2	0	2	0	0	10	28
Florez	2018	1	1	0	0	3	0	0	0	1	0	0	−5	0	0	0	0	1	3
Hamerla	2019	1	0	0	0	3	0	1	0	2	0	0	5	0	2	0	0	14	39
Kanazawa	2018	1	0	0	0	−3	0	1	1	1	0	0	−5	0	2	0	0	0	0
Ke	2019	1	0	0	0	3	0	1	0	1	0	0	3	0	2	0	0	11	31
Laukamp	2018	1	0	0	0	−3	0	0	0	0	0	0	4	2	2	0	0	6	17
Laukamp	2019	1	0	0	0	3	0	1	0	1	0	0	−5	0	2	0	0	3	8
Li	2019	1	0	0	0	3	0	1	0	1	0	0	2	2	2	0	0	12	33
Lu	2018	1	0	0	0	3	1	1	0	1	0	0	2	2	2	0	0	13	36
Morin	2019	0	0	0	0	3	1	1	0	1	0	0	3	2	2	0	0	13	36
Niu	2019	1	0	0	0	3	0	1	0	0	0	0	2	0	2	0	0	9	25
Park	2018	1	0	0	0	3	0	1	0	2	0	0	2	0	2	0	0	11	31
Speckter	2018	0	0	0	0	3	0	1	0	0	0	0	−5	0	2	0	0	1	3
Tian	2020	0	0	0	0	3	0	0	0	2	0	0	−5	0	2	0	0	2	6
Wei	2020	1	1	0	1	3	0	0	0	2	0	0	2	0	0	0	1	11	31
Yan	2017	1	0	0	0	3	0	1	0	0	0	0	2	0	2	0	0	9	25
Zhang	2019	1	0	0	0	3	0	0	0	0	0	0	−5	0	2	0	0	1	3
Zhang	2020	1	0	0	0	3	0	0	0	0	0	0	2	0	2	0	0	8	22
Zhu H	2019	0	0	0	0	−3	0	1	0	0	0	0	2	0	2	0	0	2	6
Zhu Y	2019	1	0	0	0	3	0	1	0	1	0	0	2	0	2	0	0	10	28

*RQS* radiomics quality score

**Fig. 4:**
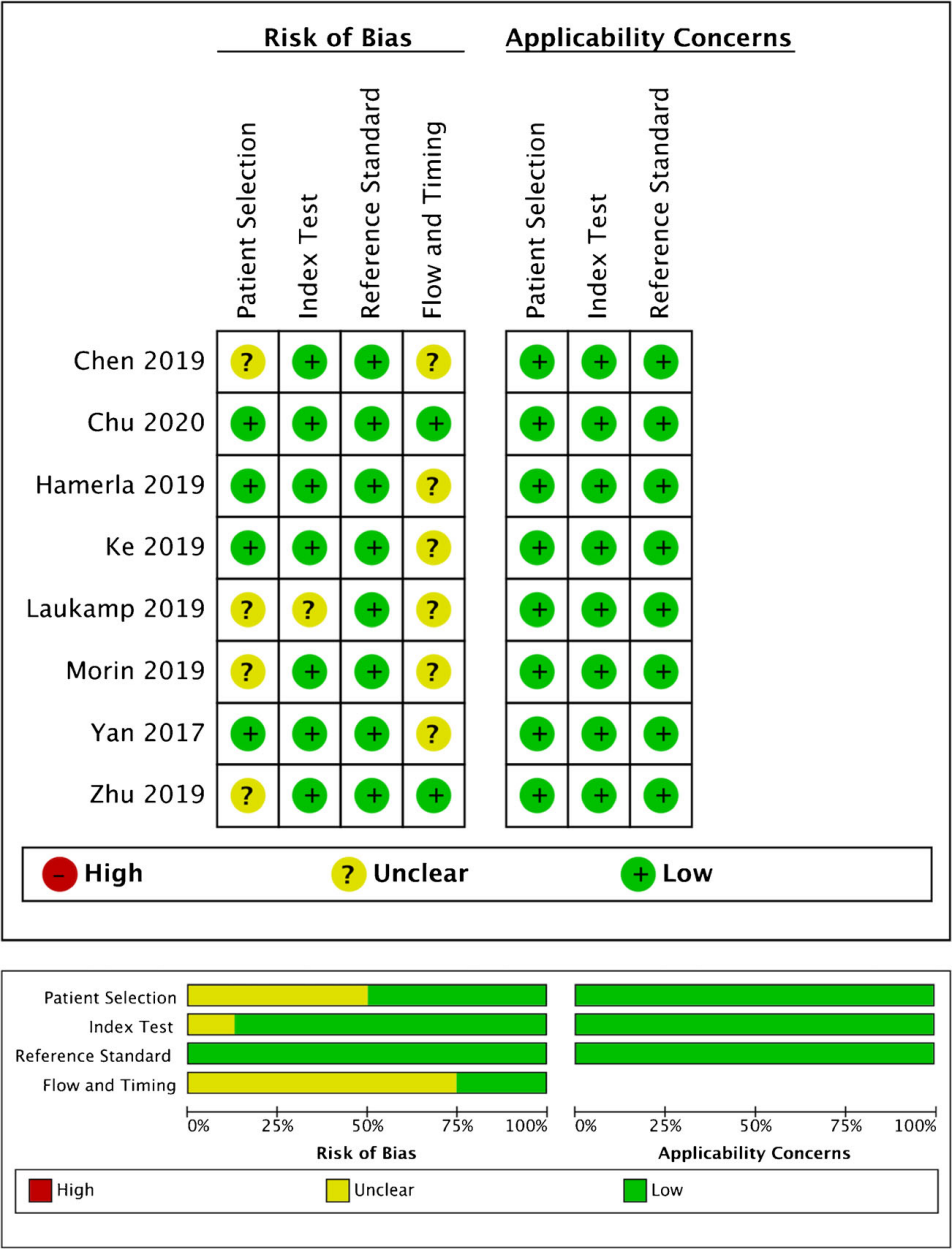
Methodological quality of the studies included in the meta-analysis according to the QUADAS 2 tool for risk of bias and applicability concerns. Green, yellow, and red circles represent low, unclear, and high risk of bias, respectively

Regarding the evaluation of the risk of bias through the QUADAS-2, the number of studies with high, unclear, and low risk of bias was respectively 0, 7, and 2, for the four domains (patient selection, index test, reference standard, and flow and timing) (Fig. 4). In particular, 4 studies scored an unclear risk of bias in the patient selection domain as the authors did not clearly report the steps of patient selection process [31, 37, 40, 47]. One study scored an unclear risk of bias in index test domain as the radiomics feature extraction was performed from both diffusion-weighted images (DWI) and apparent diffusion coefficient (ADC) maps [37]. Finally, the time elapsed between MRI and neurosurgery was not reported in 6 studies, thus scoring an unclear risk of bias in the flow and timing domain [31–45, 49]. All the studies included in the meta-analysis had low concerns regarding applicability for the three domains (patient selection, index test, and reference standard).

### Meta-analysis

**Fig. 5:**
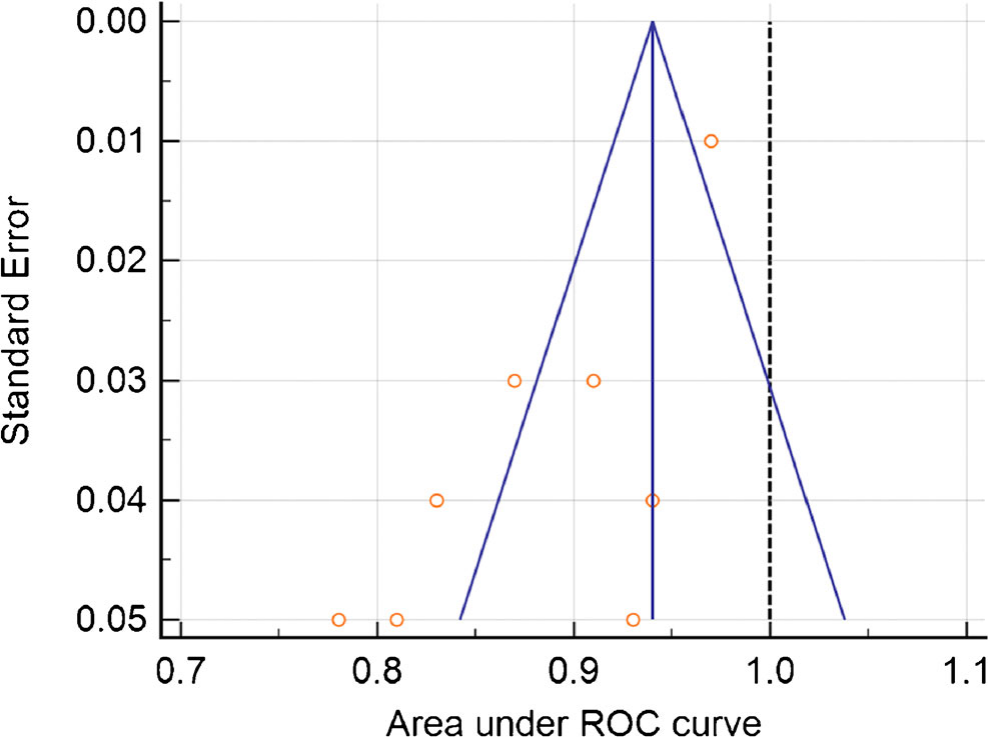
Funnel plot asymmetry test for publication bias in the literature evaluation for high-grade meningioma characterization

**Fig. 6:**
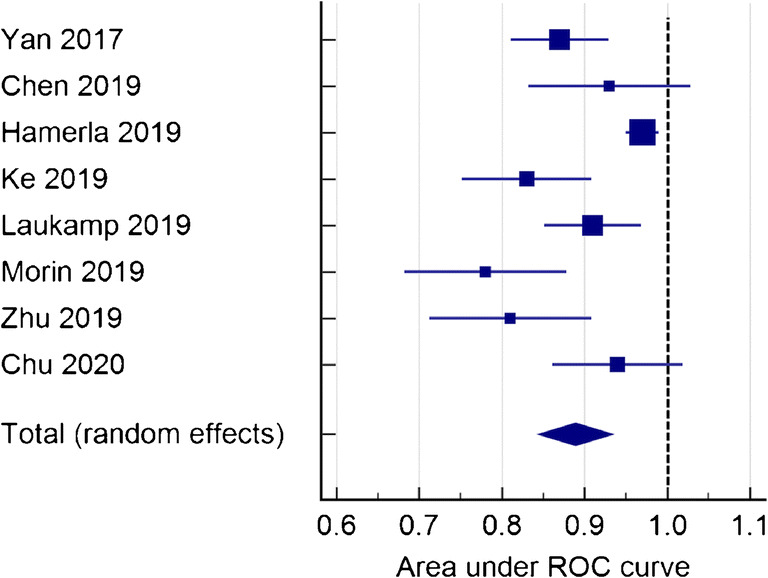
Forest plot of single studies for the pooled area under the curve (AUC) and 95% CI of high-grade meningioma characterization

The articles included in the meta-analysis are reported in Table 4. The ML models for meningioma characterization showed an overall pooled AUC = 0.88 (95% CI = 0.84–0.93) with a standard error of 0.02 (Figs. 5 and 6). Study heterogeneity was 82.5% (*p* < 0.001).

**Table 4:** Characteristics of the studies included in the meta-analysis

Paper	AUC	Low grade	High grade	Data source	Sequences	Model	Validation
Chen et al.	0.93	12	18	Single institution	CE T1	LDA	CV
Chu et al.	0.95	24	4	Single institution	CE T1	Logistic regression	Test set
Hamerla et al.	0.97	102	45	Multicenter	CE T1+others	XGBoost	CV
Ke et al.	0.83	60	19	Multicenter	CE T1+others	SVM	CV + test set
Laukamp et al.	0.91	46	25	Multicenter	CE T1+others	Logistic regression	CV
Morin et al.	0.78	67	18	Multicenter	CE T1	RF	Test set
Yan et al.	0.87	110	21	Single institution	CE T1	SVM	CV
Zhu et al.	0.82	69	13	Single institution	CE T1	LDA	CV + test set

*AUC* area under the receiver operating characteristic curve, *CE T1* contrast-enhanced T1-weighted sequence, *LDA* linear discriminant analysis, *SVM* support vector machine, *RF* random forest, *CV* cross validation

Subgroup analysis was performed to compare studies evaluating the performance of ML for meningioma characterization using patients from a single institution (*n* = 4) and from multiple centers (*n* = 4). The pooled AUC was 0.88 (95% CI = 0.84–0.92), standard error 0.02, and heterogeneity 42.17% (*p* < 0.001) in the single institution group and the pooled AUC was 0.88 (95% CI = 0.81–0.95), standard error 0.03, and heterogeneity 88.60% (*p* < 0.001) in the multi-center group.

Of the included studies, 5 used only post-contrast T1-weighted. Their pooled AUC was 0.87 (95% CI = 0.82–0.92), standard error 0.02, and heterogeneity 56.34% (*p* = 0.05). On the other hand, 3 studies also used conventional MR sequences, including T1-weighted and T2-weighted imaging, in addition to contrast-enhanced T1-weighted imaging. Their pooled AUC was 0.91 (95% CI = 0.85–0.97), standard error 0.03, and heterogeneity 85.94% (*p* < 0.001).

In a subgroup analysis based on pre-processing image type, the pooled AUC of 6 studies included in the analysis was 0.89 (95% CI = 0.85–0.94), standard error 0.02, and heterogeneity 83.01% (*p* < 0.001). The remaining studies reported an AUC value respectively of 0.93 and 0.78.

Four studies applied exclusively k-fold cross-validation for training and testing of the model. Their pooled AUC was 0.92 (95% CI = 0.88–0.97), standard error 0.02, and heterogeneity 76.52% (*p* = 0.005). The remaining studies (*n* = 4) employed a test set, in 2 cases paired with k-fold cross-validation. Their pooled AUC was 0.84 (95% CI = 0.78–0.90), standard error 0.03, and heterogeneity 62.09% (*p* < 0.005). The corresponding plots of subgroup analyses are presented in the supplementary materials.

## Discussion

Radiomics has numerous potential applications in neuroradiology and could help in obtaining additional quantitative information from routine medical images. Even though there are ongoing efforts to standardize radiomic feature extraction, their use is not yet justified outside of the research field [50]. The RQS is a recently introduced score whose aim is to evaluate the methodological quality of radiomics-based investigations. It could help identifying high-quality results among the large number of publications in this field as well as issues limiting their value and applicability. The average RQS of the articles included in our systematic review was low (6.96, 19%), reflecting a lacking overall methodological quality. This finding is in line with previous systematic reviews performing a quality assessment with the RQS tool in other fields of radiology. In detail, Ursprung et al reported a total RQS score of 3.41 ± 4.43 (9.4% average) in a review of renal cell carcinoma radiomics CT and MRI studies, Stanzione et al 7.93 ± 5.13 (23 ± 13%) for prostate MRI, and Granzier et al 20.9% for breast MRI [22, 51, 52]. Therefore, the problems affecting radiomics studies and limiting the RQS score seem to be general and not restricted to a specific application. The current situation can be at least in part explained by an exponential growth in interest and number of papers submitted using radiomics, a dynamic also experienced in the wider field of ML [7]. On the other hand, the RQS scoring system is relatively new and has been used in a limited number of occasions [18, 22, 51–53]. Therefore, further revisions and improvements after initial feedback may produce a different weighting of each item and/or modifications in the items themselves. In our review, we wish to highlight that all studies collected 0 points on items 3, 4, 10, 11, and 15. In detail, feature robustness to scanner or temporal variability was never tested, also due to the retrospective nature of all the investigations. Similarly, a prospective validation of the radiomics signature in appropriate trials was missing as well as a cost-effectiveness analysis.

Regarding the studies included in the meta-analysis, the QUADAS-2 assessment revealed an overall low risk of bias but also highlighted some critical issues. In particular, in one paper, DWI was used for feature extraction together with ADC maps [37]. As ADC maps are derived from DWI, it would be more appropriate to only use one of the two for feature extraction and probably ADC maps are preferable due to their qualitative nature. Furthermore, only two studies reported time elapsed between the MRI exam and surgery, a possible source of bias that should always be specified [11, 48] None of the articles selected scored a high risk of bias in relation to the reference standard as histopathological grading was always employed. Overall, radiomics features analyzed with a ML approach turned out to be promising for meningioma grading, with an AUC of 0.88. All the included studies used handcrafted radiomics except for Zhu et al. who employed deep learning [48]. This is understandable given that deep learning requires a large amount of data to be advantageous over other ML algorithms, often not available in this setting. Almost all studies (*n* = 7) performed a 3D segmentation of the lesion, though it is still not clear whether this approach is clearly better than 2D segmentation [48]. Only Morin et al. trained a model using radiomics features together with demographic data [40]. Despite this, its AUC value is among the lowest (0.78) suggesting that these may not be essential in the preoperative definition of meningioma grading. It is also interesting to note that most (*n* = 5) of the studies used linear ML models [11, 31, 37, 48, 49] while only one included a data augmentation technique [33].

In the subgroup analyses, AUC was higher (0.91 vs 0.87) for studies (*n* = 3) that paired T1 contrast-enhanced sequences with other sequences [11, 31, 40]. This finding supports the use of multiple imaging sequences rather than relying exclusively on T1 contrast-enhanced sequences for future investigations. Similarly, the good accuracy (AUC = 0.89) obtained by studies (*n* = 6) who included image pre-processing in their pipeline also suggests the usefulness of this step [11, 33, 37, 45, 48, 49]. While the AUC for single institution (*n* = 4) and multicenter studies was equally high (AUC = 0.88), external testing of ML models is always preferable to demonstrate their ability to generalize. Similarly, while k-fold cross-validation helps in extracting more information and reliable results from small datasets, its exclusive use may present some issues as there is no final model whose performance can be tested on unseen data. In all, 4 studies only employed cross-validation, with better results than the remaining (AUC = 0.92 vs 0.84) [11, 40, 48, 49]. Ideally, it would be preferable to use cross-validation for model tuning and initial testing followed by further assessment on new data, as done in 2 cases (AUC = 0.82 and 0.83). This approach combines the advantages of both testing strategies [48, 49].

As previously reported, the presentation of accuracy metrics in radiomics and ML studies is often inconsistent and incomplete [21]. Due to this situation, our meta-analysis could only employ AUC values as these were the most commonly reported. However, sensitivity and specificity analysis could have provided additional insights if feasible.

Indeed, ROC AUC treats sensitivity and specificity as equally important overall when averaged across all thresholds. For example, poor sensitivity could mean missed diagnosis and delayed treatment or even death, whereas poor specificity means unnecessary test. ROC AUC ignores clinical differentials in “misclassification cost” and, therefore, risks finding a new test worthless when patients and physicians would consider otherwise. ROC AUC weighs changes in sensitivity and specificity equally only where the curve slope equals one. Other points assign different weights, determined by curve shape and without considering clinically meaningful information, e.g., a 5 % improvement in sensitivity contributes less to AUC at high specificity than at low specificity. Thus, AUC can consider a test that increases sensitivity at low specificity superior to one that increases sensitivity at high specificity [54].

Greater care should be given in future research to avoid this issue, ideally confusion matrices should always be reported if possible.

The ability to distinguish low-grade from high-grade meningiomas based on preoperative MR images could influence personalized treatment decisions. In particular, in patients with meningiomas at certain locations where biopsy is difficult to obtain due to a high risk of mortality and morbidity (e.g., petroclival meningiomas), a tailored radiation treatment in the high-grade forms may be recommended [55]. Furthermore, in asymptomatic patients with small meningiomas, radiotherapy may be avoided for benign lesions, while high-grade meningiomas could undergo radiation treatment before resection [56]. Therefore, noninvasive MRI prediction of meningioma grading could address in the future small meningioma treatment strategy, also without histological confirmation. However, radiomics are not currently ready for clinical implementation due to the issues found in RQS.

Our study has some limitations that should be acknowledged. The RQS is relatively recent and a purely methodological scoring system and does not consider differences in study aim. Regarding the meta-analysis, a relatively low number of papers met the selection criteria. While the QUADAS-2 analysis presented some unclear elements, no high-risk sources of bias were identified. Study heterogeneity was high, but this is in line with other machine learning meta-analyses and diagnostic meta-analyses in general [21, 57, 58]. Finally, not all articles were specified if the WHO 2016 classification of central nervous system tumors was used. However, meningioma grading did not change substantially compared to the previous version, except for the introduction of brain invasion as a criterion for the diagnosis of grade II lesions [3].

In conclusion, radiomics studies show promising results for improving management of intracranial meningiomas, though they require more methodological rigor. The prediction of meningioma grading from preoperative brain MRI also demonstrated good results in our meta-analysis. Well-designed, prospective trials are necessary to demonstrate their validity and reporting of methods and results has to be standardized prior to their use in daily clinical practice.

## Supplementary Information

ESM 1 (PNG 550 kb)

High resolution image (TIFF 15883 kb)

ESM 2 (PNG 560 kb)

High resolution image (TIFF 16160 kb)

ESM 3 (PNG 300 kb)

High resolution image (TIFF 8787 kb)

ESM 4 (PNG 545 kb)

High resolution image (TIFF 15984 kb)

ESM 5 (DOCX 22 kb)

## Abbreviations

AUC: Area under the receiver operating characteristic curve
CI: Confidence intervals
ICC: Inter-rater correlation coefficient
ML: Machine learning
QUADAS-2: Quality Assessment of Diagnostic Accuracy Studies 2 tool
RQS: Radiomics quality score
