# Supplementary figures and images for: Meningioma MRI radiomics and machine learning: systematic review, quality score assessment, and meta-analysis
Source: Neuroradiology. 2021 Mar 2;63(8):1293–304. doi: 10.1007/s00234-021-02668-0 (PMC8295153; doi:10.1007/s00234-021-02668-0)

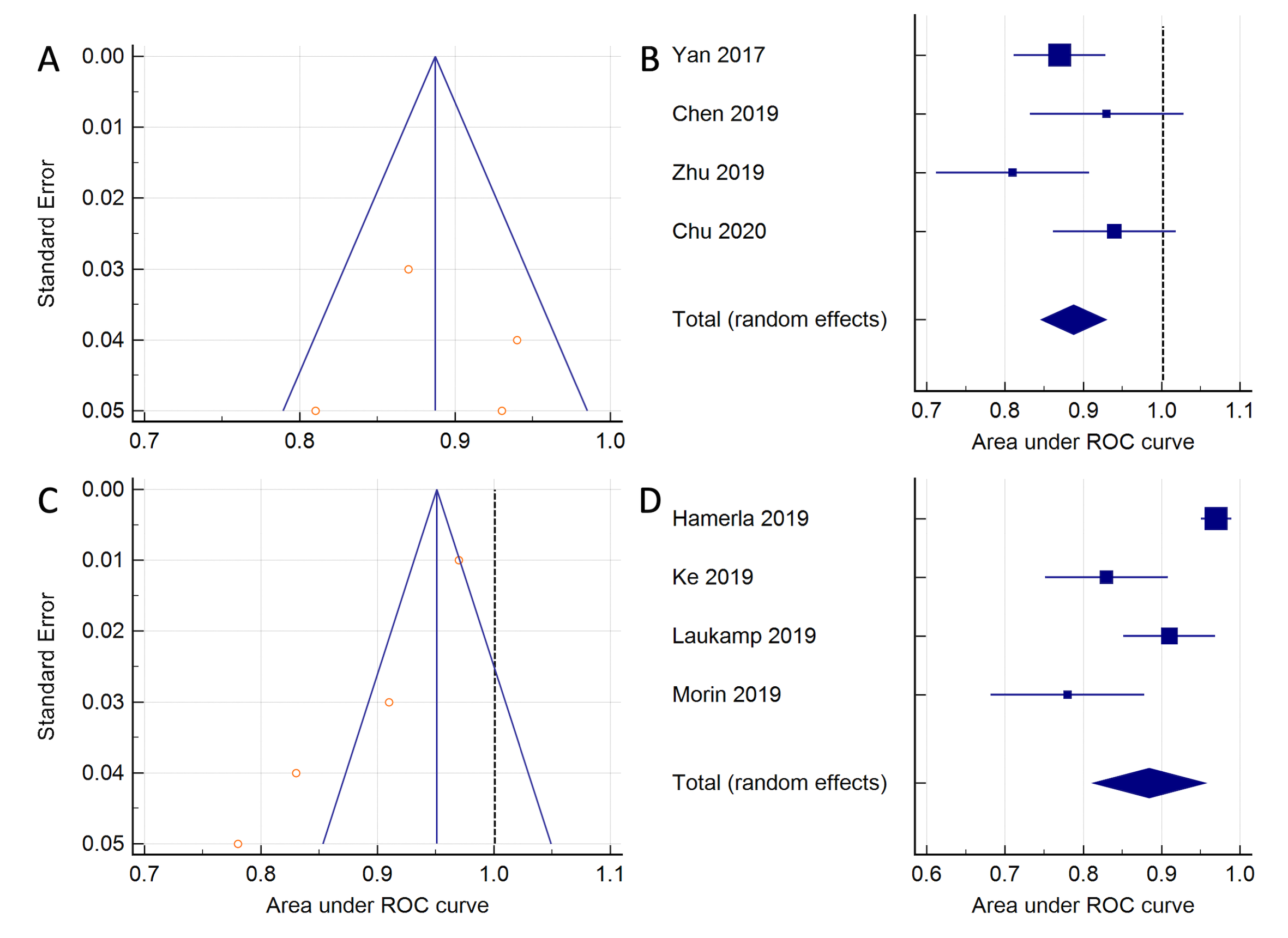

Supplement: Supplementary file 1 — (PNG 550 kb) [file 234_2021_2668_Fig7_ESM.png]

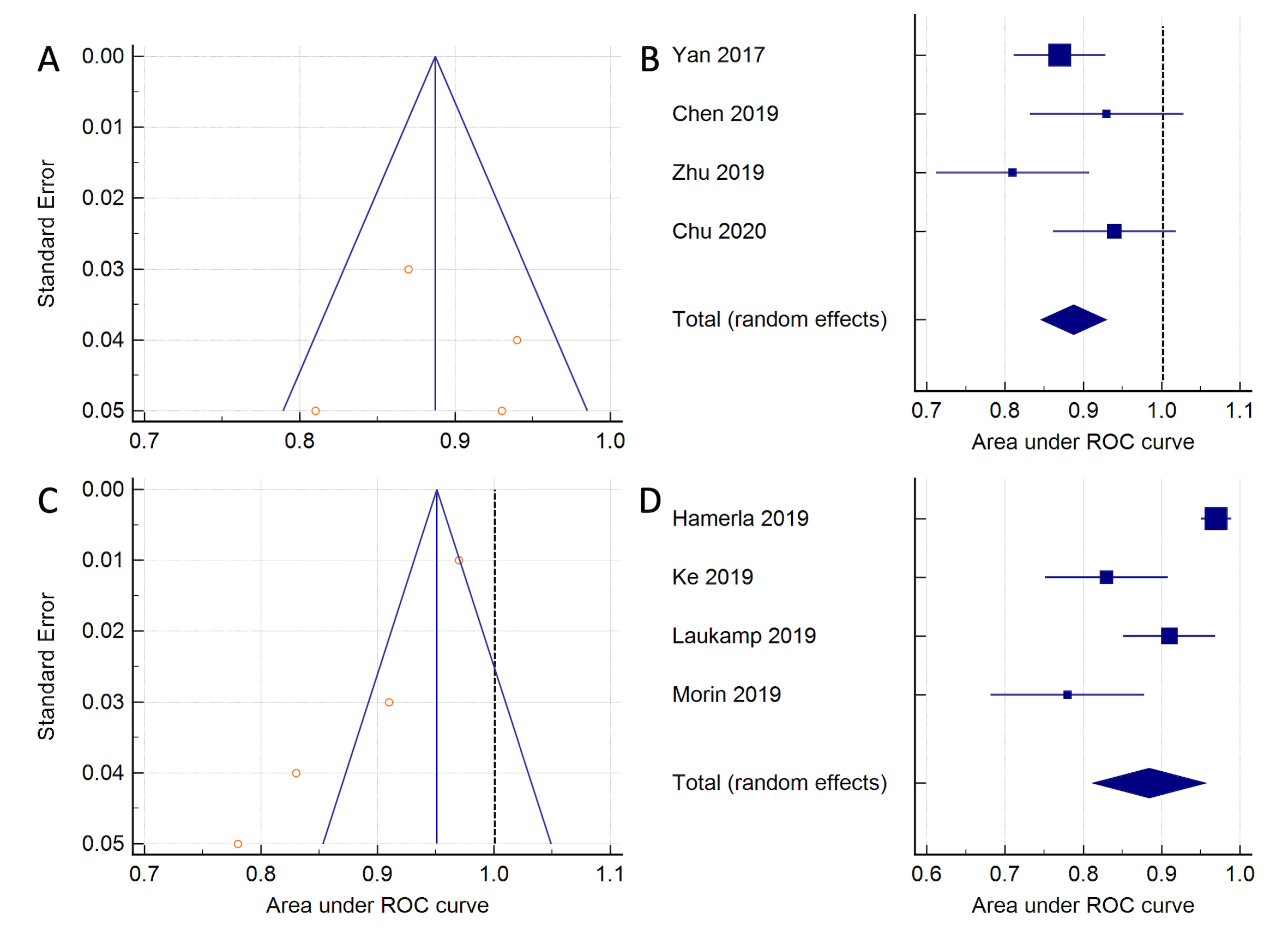

Supplement: Supplementary file 2 — High resolution image (TIFF 15883 kb) [file 234_2021_2668_MOESM1_ESM.tiff]

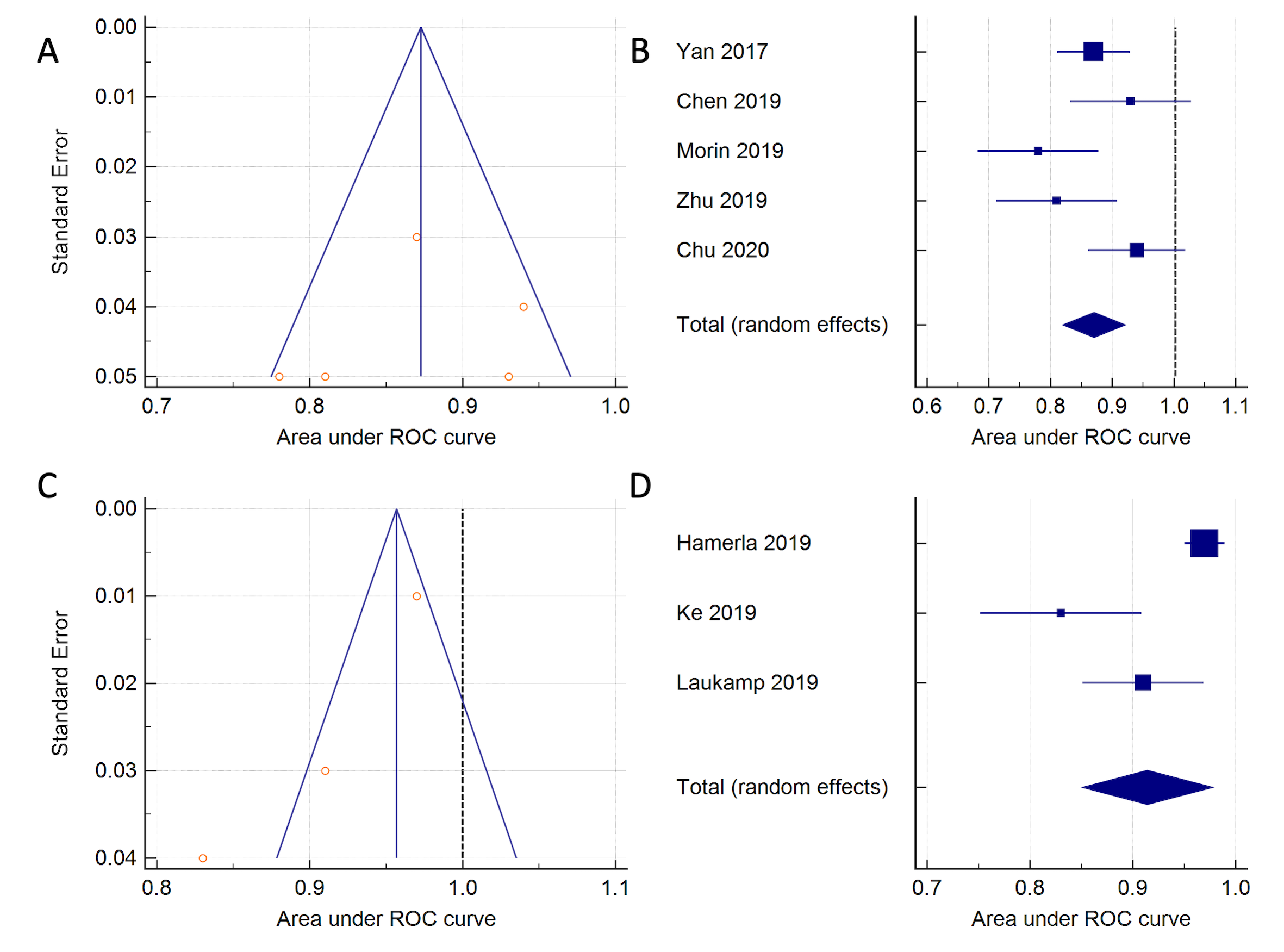

Supplement: Supplementary file 3 — (PNG 560 kb) [file 234_2021_2668_Fig8_ESM.png]

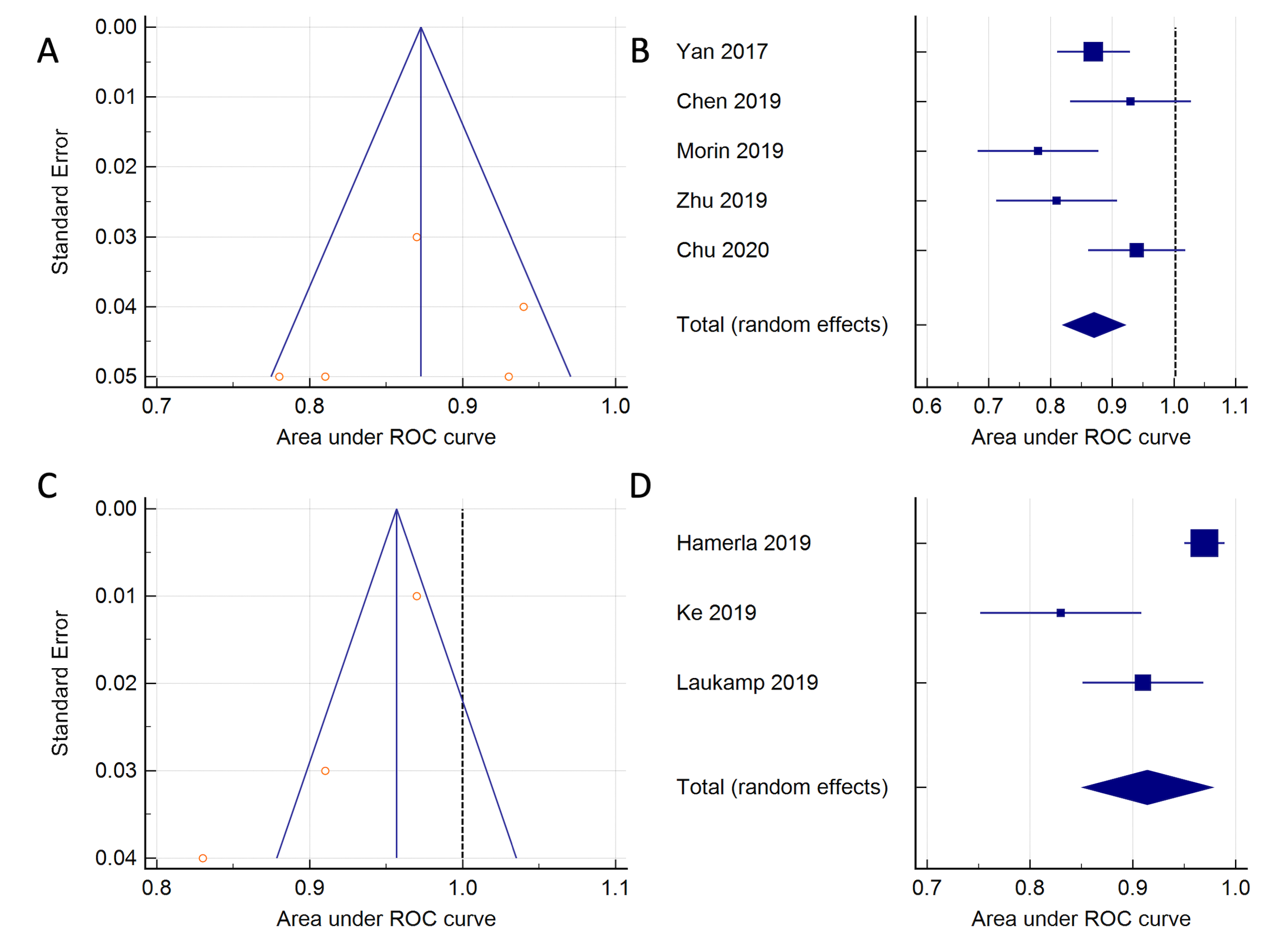

Supplement: Supplementary file 4 — High resolution image (TIFF 16160 kb) [file 234_2021_2668_MOESM2_ESM.tiff]

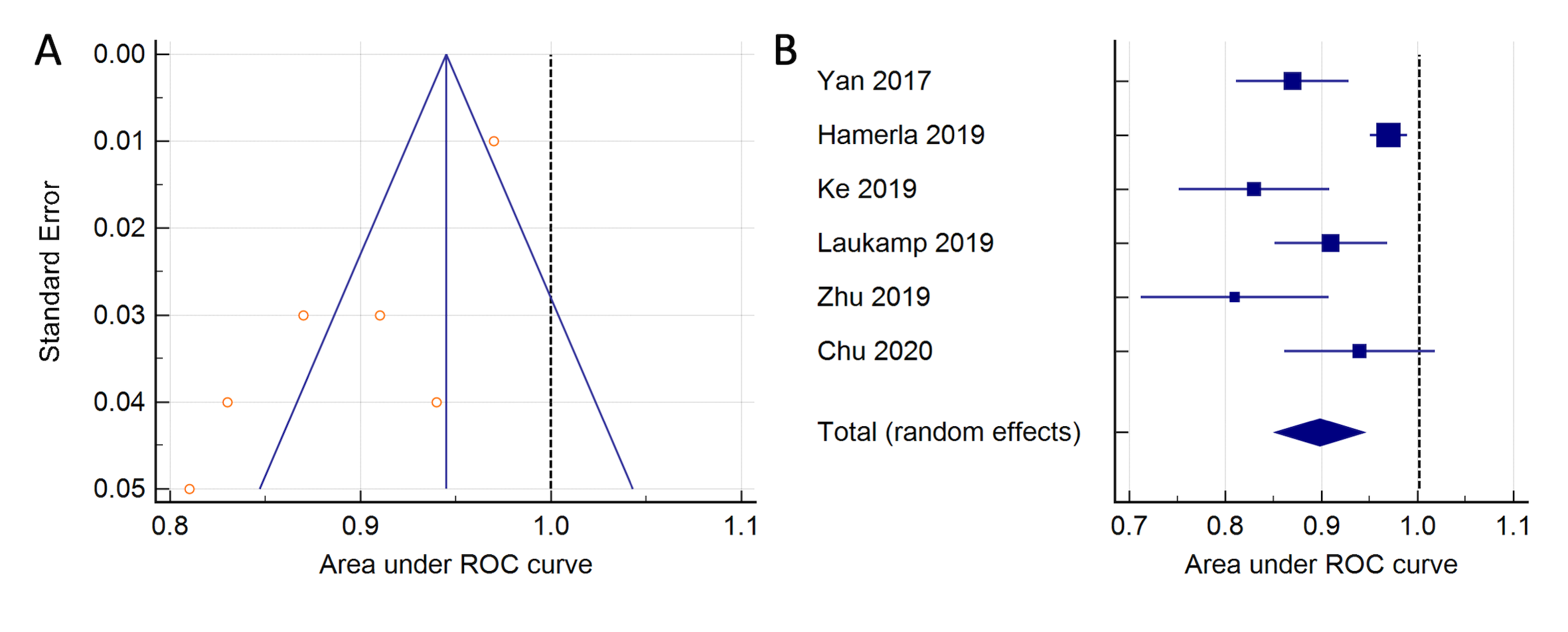

Supplement: Supplementary file 5 — (PNG 300 kb) [file 234_2021_2668_Fig9_ESM.png]

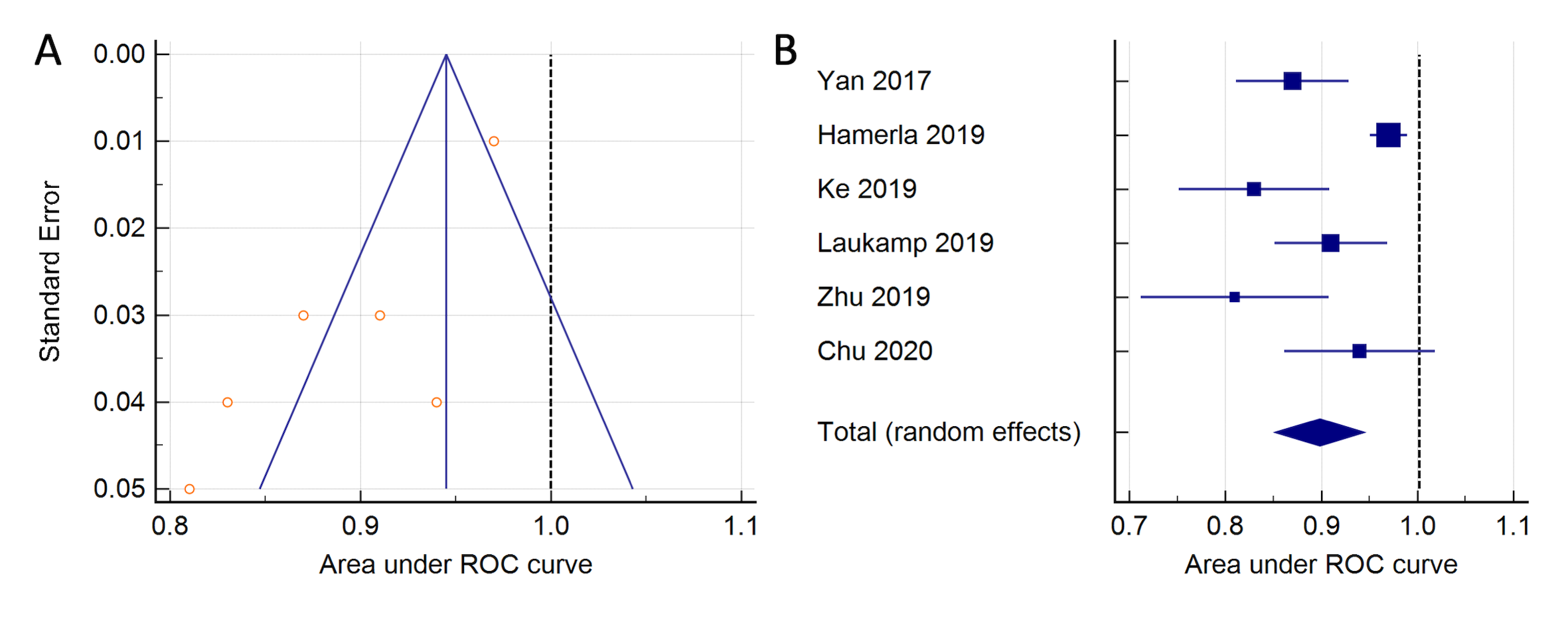

Supplement: Supplementary file 6 — High resolution image (TIFF 8787 kb) [file 234_2021_2668_MOESM3_ESM.tiff]

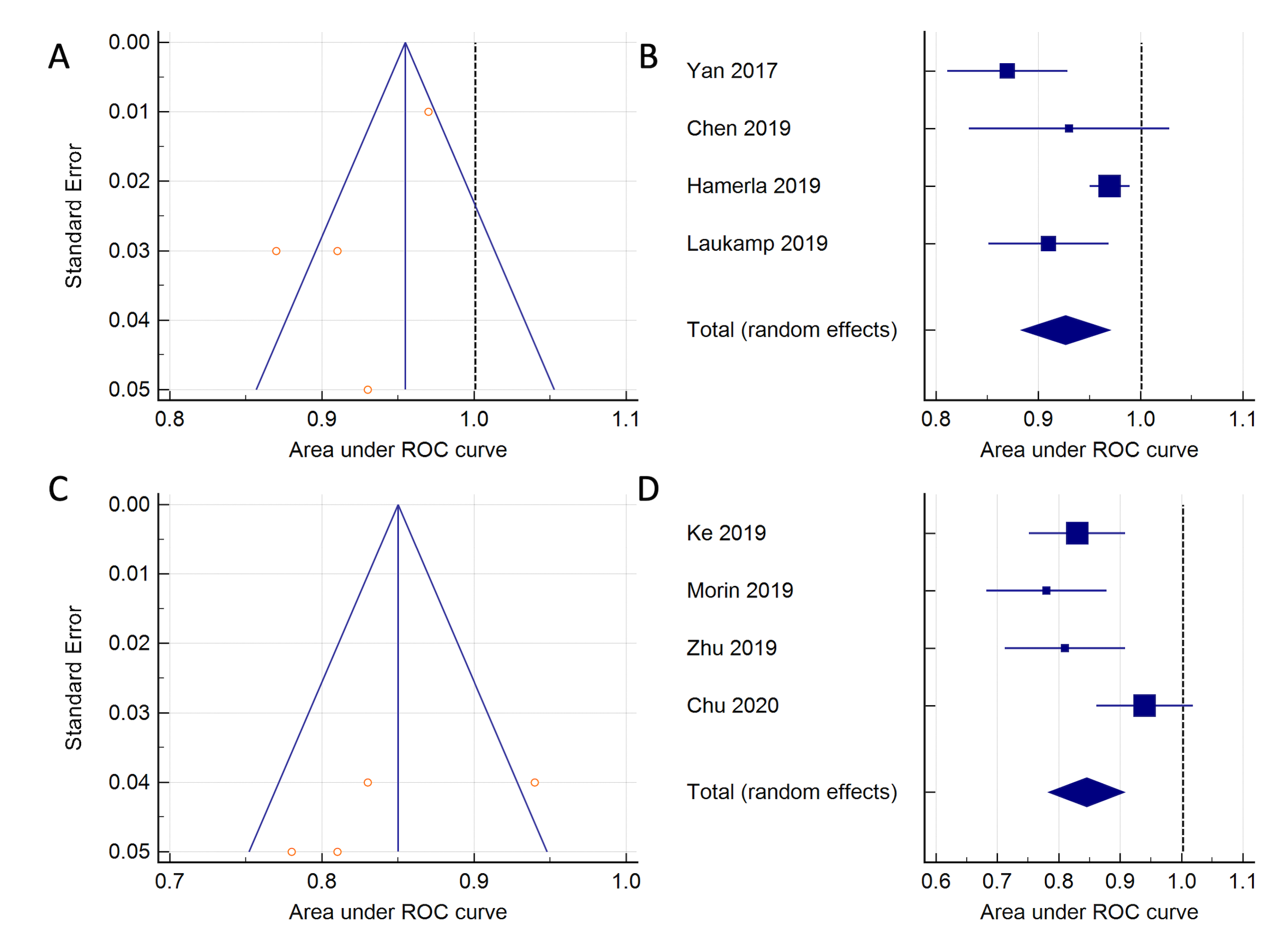

Supplement: Supplementary file 7 — (PNG 545 kb) [file 234_2021_2668_Fig10_ESM.png]

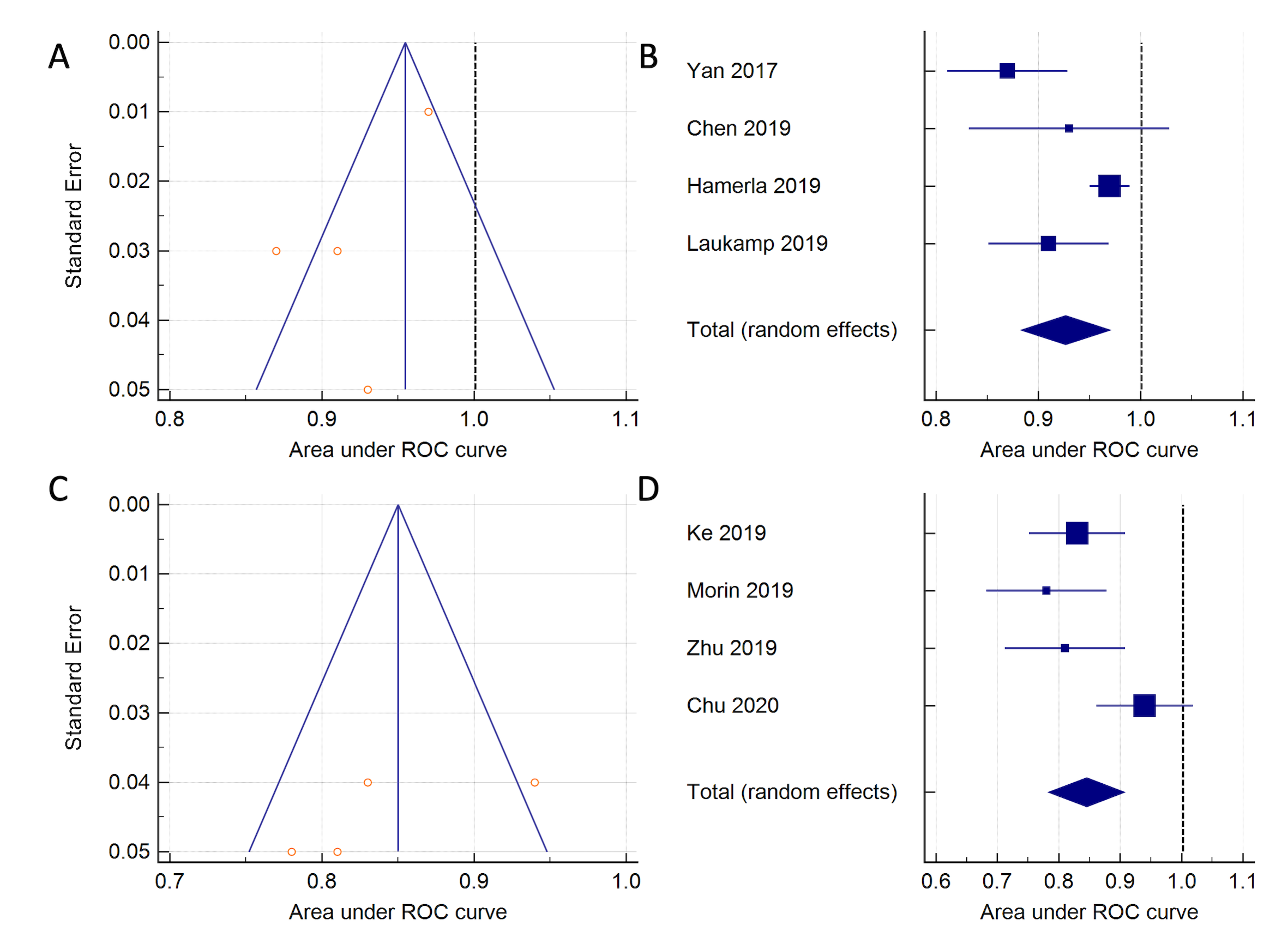

Supplement: Supplementary file 8 — High resolution image (TIFF 15984 kb) [file 234_2021_2668_MOESM4_ESM.tiff]
