# Supplementary material for: Meningioma MRI radiomics and machine learning: systematic review, quality score assessment, and meta-analysis
Source: Neuroradiology. 2021 Mar 2;63(8):1293–304. doi: 10.1007/s00234-021-02668-0 (PMC8295153; doi:10.1007/s00234-021-02668-0)
Supplement: Supplementary file 9 — (DOCX 22 kb) [file 234_2021_2668_MOESM5_ESM.docx]

**Detailed search strategy.** The full string employed for the systematic literature search, formatted for the PubMed search engine.

((((("machine learning"[MeSH Terms] OR ("machine"[All Fields] AND "learning"[All Fields])) OR "machine learning"[All Fields]) OR (("artificial intelligence"[MeSH Terms] OR ("artificial"[All Fields] AND "intelligence"[All Fields])) OR "artificial intelligence"[All Fields])) OR ("radiomic"[All Fields] OR "radiomics"[All Fields])) OR (((((((((("textural"[All Fields] OR "texturally"[All Fields]) OR "texture"[All Fields]) OR "texture s"[All Fields]) OR "textured"[All Fields]) OR "textures"[All Fields]) OR "texturing"[All Fields]) OR "texturization"[All Fields]) OR "texturize"[All Fields]) OR "texturized"[All Fields]) OR "texturizing"[All Fields])) AND (("meningioma"[MeSH Terms] OR "meningioma"[All Fields]) OR "meningiomas"[All Fields]) AND ("2000/01/01"[Date - Publication] : "2020/06/30"[Date - Publication]) AND "english"[Language]

**Supplementary table 1.** Radiomic Quality Scores for all included articles by the second reader.

| **First author** | **Year** | **Item 1** | **Item 2** | **Item 3** | **Item 4** | **Item 5** | **Item 6** | **Item 7** | **Item 8** | **Item 9** | **Item 10** | **Item 11** | **Item 12** | **Item 13** | **Item 14** | **Item 15** | **Item 16** | **RQS (total)** | **RQS (%)** |
| --- | --- | --- | --- | --- | --- | --- | --- | --- | --- | --- | --- | --- | --- | --- | --- | --- | --- | --- | --- |
| Alkubeyyer | 2020 | 0 | 0 | 0 | 0 | -3 | 0 | 0 | 0 | 2 | 0 | 0 | 2 | 0 | 2 | 0 | 0 | 6 | 17 |
| Arokiajesuprabhu | 2018 | 0 | 0 | 0 | 0 | -3 | 0 | 0 | 0 | 0 | 0 | 0 | 2 | 0 | 0 | 0 | 0 | 0 | 0 |
| Chen | 2019 | 1 | 0 | 0 | 0 | 3 | 0 | 1 | 0 | 1 | 0 | 0 | 2 | 0 | 2 | 0 | 1 | 10 | 28 |
| Chu | 2020 | 1 | 0 | 0 | 0 | 3 | 0 | 1 | 0 | 1 | 0 | 0 | 2 | 0 | 2 | 0 | 0 | 8 | 22 |
| Florez | 2018 | 1 | 1 | 0 | 0 | 3 | 0 | 0 | 0 | 1 | 0 | 0 | -5 | 0 | 2 | 0 | 0 | 6 | 17 |
| Hamerla | 2019 | 1 | 0 | 0 | 0 | 3 | 0 | 0 | 0 | 2 | 0 | 0 | 2 | 0 | 2 | 0 | 0 | 10 | 28 |
| Kanazawa | 2018 | 1 | 0 | 0 | 0 | -3 | 0 | 0 | 1 | 1 | 0 | 0 | -5 | 0 | 2 | 0 | 0 | 0 | 0 |
| Ke | 2019 | 1 | 0 | 0 | 0 | 3 | 0 | 0 | 0 | 1 | 0 | 0 | 2 | 0 | 2 | 0 | 0 | 8 | 22 |
| Laukamp | 2018 | 1 | 0 | 0 | 0 | -3 | 0 | 0 | 0 | 0 | 0 | 0 | 4 | 2 | 2 | 0 | 0 | 2 | 6 |
| Laukamp | 2019 | 1 | 0 | 0 | 0 | 3 | 0 | 0 | 0 | 1 | 0 | 0 | -5 | 0 | 2 | 0 | 0 | 6 | 17 |
| Li | 2019 | 1 | 0 | 0 | 0 | 3 | 0 | 0 | 0 | 1 | 0 | 0 | 2 | 2 | 2 | 0 | 0 | 10 | 28 |
| Lu | 2018 | 1 | 0 | 0 | 0 | 3 | 1 | 1 | 0 | 2 | 0 | 0 | 2 | 2 | 2 | 0 | 0 | 13 | 36 |
| Morin | 2019 | 0 | 0 | 0 | 0 | 3 | 1 | 1 | 0 | 2 | 0 | 0 | 3 | 2 | 2 | 0 | 0 | 13 | 36 |
| Niu | 2019 | 1 | 0 | 0 | 0 | 3 | 0 | 1 | 0 | 0 | 0 | 0 | 2 | 0 | 2 | 0 | 0 | 8 | 22 |
| Park | 2018 | 1 | 0 | 0 | 0 | 3 | 0 | 1 | 0 | 2 | 0 | 0 | 2 | 0 | 2 | 0 | 0 | 10 | 28 |
| Speckter | 2018 | 0 | 0 | 0 | 0 | 3 | 0 | 1 | 0 | 0 | 0 | 0 | -5 | 0 | 2 | 0 | 0 | 0 | 0 |
| Tian | 2020 | 0 | 0 | 0 | 0 | 3 | 1 | 0 | 0 | 2 | 0 | 0 | -5 | 0 | 2 | 0 | 0 | 1 | 3 |
| Wei | 2020 | 1 | 0 | 0 | 0 | 3 | 0 | 0 | 0 | 2 | 0 | 0 | 2 | 0 | 0 | 0 | 1 | 8 | 22 |
| Yan | 2017 | 1 | 0 | 0 | 0 | 3 | 0 | 1 | 0 | 1 | 0 | 0 | 2 | 0 | 2 | 0 | 0 | 8 | 22 |
| Zhang | 2019 | 1 | 0 | 0 | 0 | 3 | 0 | 0 | 0 | 1 | 0 | 0 | -5 | 0 | 2 | 0 | 0 | 6 | 17 |
| Zhang | 2020 | 1 | 0 | 0 | 0 | 3 | 0 | 0 | 0 | 0 | 0 | 0 | 2 | 0 | 2 | 0 | 0 | 8 | 22 |
| Zhu H | 2019 | 0 | 0 | 0 | 0 | -3 | 0 | 0 | 0 | 1 | 0 | 0 | 2 | 0 | 2 | 0 | 0 | 5 | 14 |
| Zhu Y | 2019 | 1 | 0 | 0 | 0 | 3 | 0 | 1 | 0 | 1 | 0 | 0 | 2 | 0 | 2 | 0 | 0 | 6 | 17 |

**Supplementary table 2.** Radiomic Quality Scores for all included articles by the third reader.

| **First author** | **Year** | **Item 1** | **Item 2** | **Item 3** | **Item 4** | **Item 5** | **Item 6** | **Item 7** | **Item 8** | **Item 9** | **Item 10** | **Item 11** | **Item 12** | **Item 13** | **Item 14** | **Item 15** | **Item 16** | **RQS (total)** | **RQS (%)** |
| --- | --- | --- | --- | --- | --- | --- | --- | --- | --- | --- | --- | --- | --- | --- | --- | --- | --- | --- | --- |
| Alkubeyyer | 2020 | 0 | 0 | 0 | 0 | 3 | 0 | 0 | 0 | 2 | 0 | 0 | 2 | 0 | 2 | 0 | 0 | 9 | 25 |
| Arokiajesuprabhu | 2018 | 0 | 0 | 0 | 0 | -3 | 0 | 0 | 0 | 0 | 0 | 0 | 2 | 0 | 2 | 0 | 0 | 1 | 3 |
| Chen | 2019 | 1 | 0 | 0 | 0 | 3 | 0 | 0 | 0 | 1 | 0 | 0 | 2 | 0 | 2 | 0 | 1 | 10 | 28 |
| Chu | 2020 | 1 | 0 | 0 | 0 | 3 | 0 | 0 | 0 | 2 | 0 | 0 | 2 | 0 | 2 | 0 | 0 | 10 | 28 |
| Florez | 2018 | 1 | 0 | 0 | 0 | 3 | 0 | 0 | 0 | 2 | 0 | 0 | 2 | 0 | 2 | 0 | 0 | 10 | 28 |
| Hamerla | 2019 | 1 | 0 | 0 | 0 | 3 | 0 | 0 | 1 | 1 | 0 | 0 | -5 | 0 | 2 | 0 | 0 | 3 | 8 |
| Kanazawa | 2018 | 1 | 0 | 0 | 0 | 3 | 0 | 0 | 0 | 2 | 0 | 0 | 2 | 0 | 2 | 0 | 0 | 10 | 28 |
| Ke | 2019 | 1 | 0 | 0 | 0 | -3 | 0 | 0 | 0 | 0 | 0 | 0 | 2 | 2 | 2 | 0 | 0 | 4 | 11 |
| Laukamp | 2018 | 1 | 0 | 0 | 0 | 3 | 0 | 0 | 0 | 2 | 0 | 0 | 2 | 0 | 2 | 0 | 0 | 10 | 28 |
| Laukamp | 2019 | 1 | 0 | 0 | 0 | 3 | 1 | 0 | 0 | 2 | 0 | 0 | 2 | 2 | 2 | 0 | 0 | 13 | 36 |
| Li | 2019 | 1 | 0 | 0 | 0 | 3 | 1 | 0 | 0 | 2 | 0 | 0 | 2 | 2 | 2 | 0 | 0 | 13 | 36 |
| Lu | 2018 | 0 | 0 | 0 | 0 | 3 | 1 | 0 | 0 | 2 | 0 | 0 | 3 | 2 | 2 | 0 | 0 | 13 | 36 |
| Morin | 2019 | 1 | 0 | 0 | 0 | 3 | 0 | 0 | 0 | 0 | 0 | 0 | 2 | 0 | 2 | 0 | 0 | 8 | 22 |
| Niu | 2019 | 1 | 0 | 0 | 0 | 3 | 0 | 0 | 0 | 2 | 0 | 0 | 2 | 0 | 2 | 0 | 0 | 10 | 28 |
| Park | 2018 | 0 | 0 | 0 | 0 | 3 | 0 | 0 | 0 | 0 | 0 | 0 | -5 | 0 | 2 | 0 | 0 | 0 | 0 |
| Speckter | 2018 | 0 | 0 | 0 | 0 | 3 | 1 | 0 | 0 | 1 | 0 | 0 | -5 | 0 | 2 | 0 | 0 | 2 | 6 |
| Tian | 2020 | 1 | 0 | 0 | 0 | 3 | 0 | 0 | 0 | 2 | 0 | 0 | 2 | 0 | 2 | 0 | 0 | 10 | 28 |
| Wei | 2020 | 1 | 0 | 0 | 0 | 3 | 0 | 0 | 0 | 2 | 0 | 0 | 2 | 0 | 2 | 0 | 0 | 10 | 28 |
| Yan | 2017 | 0 | 0 | 0 | 0 | 3 | 0 | 0 | 0 | 1 | 0 | 0 | 2 | 0 | 2 | 0 | 0 | 8 | 22 |
| Zhang | 2019 | 1 | 0 | 0 | 0 | 3 | 0 | 0 | 0 | 2 | 2 | 0 | 3 | 0 | 2 | 0 | 0 | 13 | 36 |
| Zhang | 2020 | 1 | 0 | 0 | 0 | 3 | 0 | 0 | 0 | 2 | 0 | 0 | 2 | 0 | 2 | 0 | 0 | 10 | 28 |
| Zhu H | 2019 | 1 | 0 | 0 | 0 | 3 | 1 | 0 | 0 | 2 | 2 | 0 | 2 | 0 | 2 | 0 | 0 | 13 | 36 |
| Zhu Y | 2019 | 1 | 0 | 0 | 0 | 3 | 0 | 0 | 0 | 1 | 0 | 0 | 2 | 0 | 2 | 0 | 0 | 9 | 25 |

**Supplementary figure 1**

1. Funnel plot asymmetry test for publication bias in the single center study subgroup. Each dot represents a study; the y-axis represents study precision (standard error of effect size) and the x-axis shows the effect size. Large studies appear toward the top of the graph and tend to cluster near the mean effect size. Small studies appear toward the bottom of the graph and are dispersed across a range of values since there is more sampling variation in effect size estimates. The outer dashed lines indicate the triangular region within which 95% of studies are expected to lie in the absence of both biases and heterogeneity. The dashed line marks the AUC upper limit.
2. Forest plot of single center studies for the pooled area under the curve (AUC) and 95% CI of meningioma grading. Horizontal lines represent 95% confidence interval of the point estimates. Each solid box represents AUC of individual studies, and the size of the box indicates the study size. The diamond means the pooled AUC of all studies. The dashed line marks the AUC upper limit.
3. Funnel plot asymmetry test for publication bias in the multicenter study subgroup. The outer dashed lines indicate the triangular region within which 95% of studies are expected to lie in the absence of both biases and heterogeneity. The dashed line marks the AUC upper limit.
4. Forest plot of multicenter studies for the pooled area under the curve (AUC) and 95% CI of meningioma grading.

**Supplementary figure 2**

1. Funnel plot asymmetry test for publication bias in the study subgroup using only contrast-enhanced T1-weighted sequence.
2. Forest plot of studies using only contrast-enhanced T1-weighted sequence for the pooled area under the curve (AUC) and 95% CI of meningioma grading.
3. Funnel plot asymmetry test for publication bias in the study subgroup using contrast-enhanced T1-weighted together with other sequences.
4. Forest plot of studies using contrast-enhanced T1-weighted together with other sequences for the pooled area under the curve (AUC) and 95% CI of meningioma grading.

**Supplementary figure 3**

1. Funnel plot asymmetry test for publication bias in the study subgroup performing image pre-processing.
2. Forest plot of studies performing image pre-processing for the pooled area under the curve (AUC) and 95% CI of meningioma grading.

**Supplementary figure 4**

1. Funnel plot asymmetry test for publication bias in the study subgroup using cross validation.
2. Forest plot of studies using cross validation for the pooled area under the curve (AUC) and 95% CI of meningioma grading.
3. Funnel plot asymmetry test for publication bias in the study subgroup using a test set.
4. Forest plot of studies using a test set for the pooled area under the curve (AUC) and 95% CI of meningioma grading.
